# An explorative study on deep profiling of peripheral leukocytes to identify predictors for responsiveness to anti-tumour necrosis factor alpha therapies in ankylosing spondylitis: natural killer cells in focus

**DOI:** 10.1186/s13075-018-1692-y

**Published:** 2018-08-29

**Authors:** Ursula Schulte-Wrede, Till Sörensen, Joachim R. Grün, Thomas Häupl, Heike Hirseland, Marta Steinbrich-Zöllner, Peihua Wu, Andreas Radbruch, Denis Poddubnyy, Joachim Sieper, Uta Syrbe, Andreas Grützkau

**Affiliations:** 1 German Rheumatism Research Center Berlin (DRFZ), an Institute of the Leibniz-Association, Immune Monitoring Core Facility, Charitéplatz 1, 10117 Berlin, Germany; 2 Department of Rheumatology and Clinical Immunology, Charité - Universitätsmedizin Berlin, Berlin, Germany; 3 Department of Gastroenterology, Infectiology and Rheumatology, Charité - Universitätsmedizin Berlin, Berlin, Germany; 4 German Rheumatism Research Center Berlin (DRFZ), an Institute of the Leibniz-Association, Bioinformatics Group, Berlin, Germany; 5 German Rheumatism Research Center Berlin (DRFZ), an Institute of the Leibniz-Association, Cell Biology Group, Berlin, Germany; 6 German Rheumatism Research Center Berlin (DRFZ), an Institute of the Leibniz-Association, Epidemiology Unit, Berlin, Germany

**Keywords:** Ankylosing spondylitis, Etanercept, CD8^+^ NK cells, TNF-alpha blocker, Predictive biomarker

## Abstract

**Background:**

Therapeutic targeting of tumour necrosis factor (TNF)-α is highly effective in ankylosing spondylitis (AS) patients. However, since one-third of anti-TNF-treated AS patients do not show an adequate clinical response there is an urgent need for new biomarkers that would aid clinicians in their decision-making to select appropriate therapeutic options. Thus, the aim of this explorative study was to identify cell-based biomarkers in peripheral blood that could be used for a pre-treatment stratification of AS patients.

**Methods:**

A high-dimensional, multi-parametric flow cytometric approach was applied to identify baseline predictors in 31 AS patients before treatment with the TNF blockers adalimumab (TNF-neutralisation) and etanercept (soluble TNF receptor).

**Results:**

As the major result, the frequencies of natural killer (NK) cells, and in particular CD8-positive (CD8^+^) NK cell subsets, were most predictive for therapeutic outcome in AS patients. While an inverse correlation between classical CD56^+^/CD16^+^ NK cells and reduction of disease activity was observed, the CD8^+^ NK cell subset behaved in the opposite direction. At baseline, responders showed significantly increased frequencies of CD8^+^ NK cells compared with non-responders.

**Conclusions:**

This is the first study demonstrating that the composition of the NK cell compartment has predictive power for prediction of therapeutic outcome for anti-TNF-α blockers, and we identified CD8^+^ NK cells as a potential new player in the TNF-α-driven chronic inflammatory immune response of AS.

**Electronic supplementary material:**

The online version of this article (10.1186/s13075-018-1692-y) contains supplementary material, which is available to authorized users.

## Background

Ankylosing spondylitis (AS) is a multifactorial chronic inflammatory rheumatic disease belonging to the group of rheumatic diseases known as spondyloarthritis (SpA) which primarily affects the axial skeleton [1]. AS has a prevalence of about 1.43 million in the European population [2] with an onset in adolescence [3] and a two-times higher occurrence in men than women [4]. The pathogenesis of AS is still obscure; it is assumed that AS is mainly caused by both genetic factors, which implies the expression of the major histocompatibility complex (MHC) I antigen human leukocyte antigen B27 (HLA-B27) [5], and also by environmental factors such as enterobacterial antigens [6]. To alleviate the axial symptoms of AS patients, non-steroidal anti-inflammatory drugs (NSAIDs) are delivered as first-line therapy. Anti-tumour necrosis factor (TNF) blocking therapy is applied only in patients with constantly high disease activity who are non-responsive to conventional NSAID treatment [7, 8]. At present, there are five anti-TNF-α agents approved for the treatment of AS: infliximab [9], a monoclonal chimeric antibody; etanercept, a soluble human TNF receptor (sTNFR)2 fusion protein [10]; adalimumab, a humanised monoclonal antibody [11]; golimumab, a fully human monoclonal antibody [12]; and certoluzimab, a Fab fragment of a humanised monoclonal antibody [13]. Most of these biologics are also successfully administered in rheumatoid arthritis (RA), psoriasis (Pso), juvenile inflammatory arthritis (JIA), and inflammatory bowel disease (IBD). The biological functions of TNF-α are mediated by binding to the membrane receptors TNFR1 (p55) or TNFR2 (p75). While TNFR1 is ubiquitously expressed in all lymphoid and myeloid immune cells and body cells [14], the expression of TNFR2 is mainly restricted to T cells [15] and natural killer (NK) cells [16]. In addition, TNFR2 can be found to be expressed in endothelial and mesenchymal cells, cells of the central nervous system (CNS), oligodendrocytes and cardiac myocytes [17], and a few other cell types [18]. According to their functions, TNFR1 is primarily associated with pro-apoptotic processes, while TNFR2 is responsible for processes ensuring survival of cells [19].

Although targeting of TNF-α is very effective in AS, around one-third of treated patients show only a poor response that can be partly attributed to the development of anti-drug antibodies (ADAb) resulting in reduced bioavailability [20]. The most likely precondition for swapping to another anti-TNF agent is partial or entire failure of effectiveness along with side effects [21]. With respect to these adverse reactions and the high costs of anti-TNF agents leading to high economic burden for the health care systems, it is desirable to stratify patients according to treatment predictors prior to biological therapy. Various demographic and clinical parameters such as high baseline disease activity, short disease duration, young age, male sex, and presence of HLA-B27 have been shown to correlate with adequate clinical short- and long-term response [22–26]. In addition, modern imaging techniques, such as magnetic resonance imaging (MRI), are used to correlate bone and tissue destruction with treatment response [27]. However, these techniques are time consuming and expensive when used as a standard pre-treatment assessment. Another level of therapy response prediction was investigated when responsiveness to anti-TNF agents was related to the presence of different TNF-α genotypes. It was reported that patients with TNF-α–308 G/G, –857 C/C, or –1031 T/T genotypes showed a better response to anti-TNF agents than patients without these polymorphisms [28, 29]. Apart from these observations, there are no reliable predictive biomarkers for anti-TNF responsiveness in AS. Using a multi-parametric flow cytometric approach, we aimed to identify cell-based biomarkers in the peripheral blood of AS patients that are able to predict a successful therapeutic response to TNF inhibitors before starting therapy. As a result, we found that a low pre-treatment frequency of a CD8-expressing subpopulation of NK cells is associated with a lack of therapeutic response.

## Methods

### Subjects

#### Ethics statement

The study was performed in accordance with the 1964 Declaration of Helsinki and approved by the Charité University Medicine ethics committee I of Charité Campus Mitte. All patients provided written informed consent to participate in the study. Furthermore, we declare that this manuscript contains no information or images that could lead to identification of a study participant.

A total of 31 AS patients (22 male, 9 female) of whom 81% were positive for HLA-B27, recruited from the rheumatology outpatient clinics of the Charité, and 10 healthy controls (HC; 7 male, 3 female) participated in the study. The patients had an average age of 38 ± 10.2 years and the HC averaged 34 ± 10.7 years. All patients fulfilling the modified New York criteria [30] and who were eligible for anti-TNF inhibitor treatment because of persistently high disease activity (Bath Ankylosing Spondylitis Disease Activity Index (BASDAI) > 4) despite treatment with NSAIDs or who were unable to take NSAIDs due to contraindications were included in the study (Table 1). Disease activity was assessed according to the BASDAI index consisting of a score range from 0 (no symptoms) to 10 (high disease activity). The mean baseline BASDAI prior to TNF inhibitor therapy was 6.2 ± 1.3 (Table 1).

**Table 1:** Demographic and disease characteristics of AS patients treated with ETN and ADA, respectively

	ADA- and ETN-treated AS patients (*n* = 31)	ETN-treated AS patients (*n* = 15)	ADA-treated AS patients (*n* = 16)
Demographics			
Sex (M:F)	22:9	11:4	11:5
Age (years)	38 ± 10.2	37.7 ± 11.5	38.3 ± 9.2
Disease status			
DD (months)	140.8 ± 117.9	145.1 ± 135.4	136.8 ± 103.8
BASDAI at baseline	6.2 ± 1.3	6.2 ± 1.5	6.2 ± 1.2
BASDAI red. (%)	47.6 ± 31.3	51.1 ± 31.7	44.3 ± 31.6
BASDAI assessment (months)	3.1 ± 1.4	3 ± 1	3.2 ± 1.6
BASDAI50 (R:NR)	19:12	10:5	9:7
CRP (mg/dl)	1.5 ± 1.7	1.3 ± 1.1	1.6 ± 2.2
HLA-B27-positive (%)	81	87	75
ESR (mm/h)	33.2 ± 21.9	31.7 ± 22.3	34.6 ± 22.3

Results are displayed as mean ± SD unless otherwise indicated

*ADA* adalimumab, *AS* ankylosing spondylitis, *BASDAI* Bath Ankylosing Disease Activity Index, *BASDAI red.* percental BASDAI reduction after 1–6 month of therapy, *BASDAI50* percental BASDAI reduction according to an improvement of 50%, *CRP* C-reactive protein, *DD* disease duration, *ESR* erythrocyte sedimentation rate, *ETN* etanercept, *F* female, *HLA* human leukocyte antigen, *M* male, *NR* non-responder, *R* responder

Prior to the start of TNF inhibitor therapy, 10 ml heparinised blood was taken to perform flow cytometric analysis. Fifteen patients were treated with etanercept (Enbrel; Amgen, and Pfizer) and 16 patients with adalimumab (Humira; AbbVie Inc.). The BASDAI score was obtained at baseline and at follow-up visits [31]. The response to treatment was assessed between 1 and 6 months after the start of therapy and defined as a 50% BASDAI reduction (BASDAI50 response) relative to baseline BASDAI (Additional file 1: Table S1).

### Blood sample preparation, antibody staining, and flow cytometry measurement

Blood sample preparation and antibody staining procedures were as described previously [32]. Cells obtained from the blood of patients prior to treatment were stained for 50 different surface antigens in a seven-colour staining combined to 10 tubes (Table 2). After staining, cells were fixed with 1% paraformaldehyde and analysed within 24 h. We did not include a live/dead cell staining, but cell debris, erythrocytes, and thrombocytes were excluded according to their SSC/FSC characteristics.

**Table 2:** Staining matrix showing antibodies and their corresponding fluorochrome conjugates measured in ten separate staining tubes

Fluorochrome	T1	T2	T3	T4	T5	T6	T7	T8	T9	T10
Pacific Blue	CD3	CD3	CD3	CD3	CD3	CD3	CD3	CD3	CD3	CD3
FITC	CD27	CD64	CD244	CD35	CD46	CD45RA	BDCA2	CD138	CD134	CD28
PE	IgD/ CD14/ CD56	CD33/ NKG2D	CD163/ CRTH-2	CD119	CD88	CXCR4	CD1c	CD38	ICOS	CD31
PE-Cy5	CD45RA	HLA-DR	CD128b	CD107a	CD21	CD62L	HLA-DR	HLA-DR	CD154	CD45RA
PE-Cy7	CD8	CD56/ CD14	CD14	CD14	CD14	CCR7	CD14	CD69	CD69	CD69
APC	CD19	CD32	CCR2	CD120b	CD55	CXCR3	CD11c	CD20	CD25	CD152
APC-Cy7	CD4/ CD16	CD4/ CD16	CD4/ CD16	CD4/ CD16	CD4/ CD16	CD4/ CD16	CD19	CD19	CD4/ CD16	CD4/ CD16

T1–T10 represents the respective staining tubes

Data acquisition was accomplished with a FACSCanto™ II Flow Cytometer (BD Biosciences, USA) with an average cell count of one million cells per sample. To warrant reproducibility and to survey the instruments’ performance, a BD™ Cytometer Setup and Tracking Beads were regularly used before each measurement. In addition, we have always monitored the quality of antibody staining directly after data acquisition by monitoring each individual fluorescence channel used for each particular staining tube. For this 20,000 randomly selected events were plotted. Samples which did not pass this quality check were excluded from further analysis.

### Data analysis and statistical analysis

Two different software tools were applied to analyse the complex datasets generated by this unsupervised flow cytometry approach, which is based on both manual and automatic bioinformatic strategies identify potential candidate phenotypes. In the first approach, the relevant statistics such as mean fluorescence intensities (MFIs) and absolute cell numbers of manually analysed data were transferred as a comma-separated value (CSV) file format to an Access database as shown previously [32]. This primary data analysis including colour compensation and gate setting was performed by FACSDIVA v6.0 software (BD Biosciences, USA). The second approach utilised the automated classification algorithm immunoClust, which processes uncompensated raw data and therefore excludes any operator-dependent gating or compensation artefacts [33]. Population clustering and comparative meta-clustering of immunoClust assume finite mixture models and use Expectation Maximisation (EM)-iterations with an integrated classification likelihood (ICL) criterion to stabilise the number of reasonable clusters. For meta-clustering, a probability measure on Gaussian distributions was invented, which is based on the Bhattacharyya Coefficients. Meta-clusters were manually annotated and classified.

Linear regression and receiver operating characteristic (ROC) analysis performed with Prism 5 (GraphPad Software, Inc.) was used to elucidate associations between candidate markers and clinical parameters. For statistical data analysis, the Welch corrected *t* test was used where *p* values < 0.05 were determined as statistically significant.

## Results

### Patient baseline characteristics and their clinical responses

The study design encompassed 31 AS patients with high disease activity indicated by a baseline BASDAI of 6.2 ± 1.3 before treatment with adalimumab (ADA; *n* = 16) or etanercept (ETN; *n* = 15). The patient demographic and baseline clinical characteristics are summarised in Table 1 and showed no significant differences between the ADA- and ETN-treated patients.

The average BASDAI assessment date was 3.1 ± 1.4 month after the start of treatment. After 1 to 6 months of treatment, the relative reduction of disease activity assessed by BASDAI was 51.1 ± 31.7% for ETN-treated patients and 44.3 ± 31.6% for patients who received ADA. According to the BASDAI response criteria, five patients in the ETN group and seven patients in the ADA group failed to respond (Additional file 1: Table S1).

Clinical parameters available at baseline, such as disease duration, baseline BASDAI, C-reactive peptide (CRP) levels, and erythrocyte sedimentation rate (ESR), as well as the expression of HLA-B27, allowed no discrimination between patients who would respond to TNF-α blockers and those who would fail.

For data analysis, we have applied an unsupervised, automated cell clustering approach, immunoClust [33], to identify potential immunophenotypic parameters that enable classification of AS patients into responders (R) and non-responders (NR) prior to anti-TNF treatment. Since the freshly obtained patient blood was immediately processed we have not performed dead cell staining, but we gated for live cells according to cell size and granularity defined by forward scatter (FSC) and sideward scatter (SSC) characteristics, respectively.

**Fig. 1:**
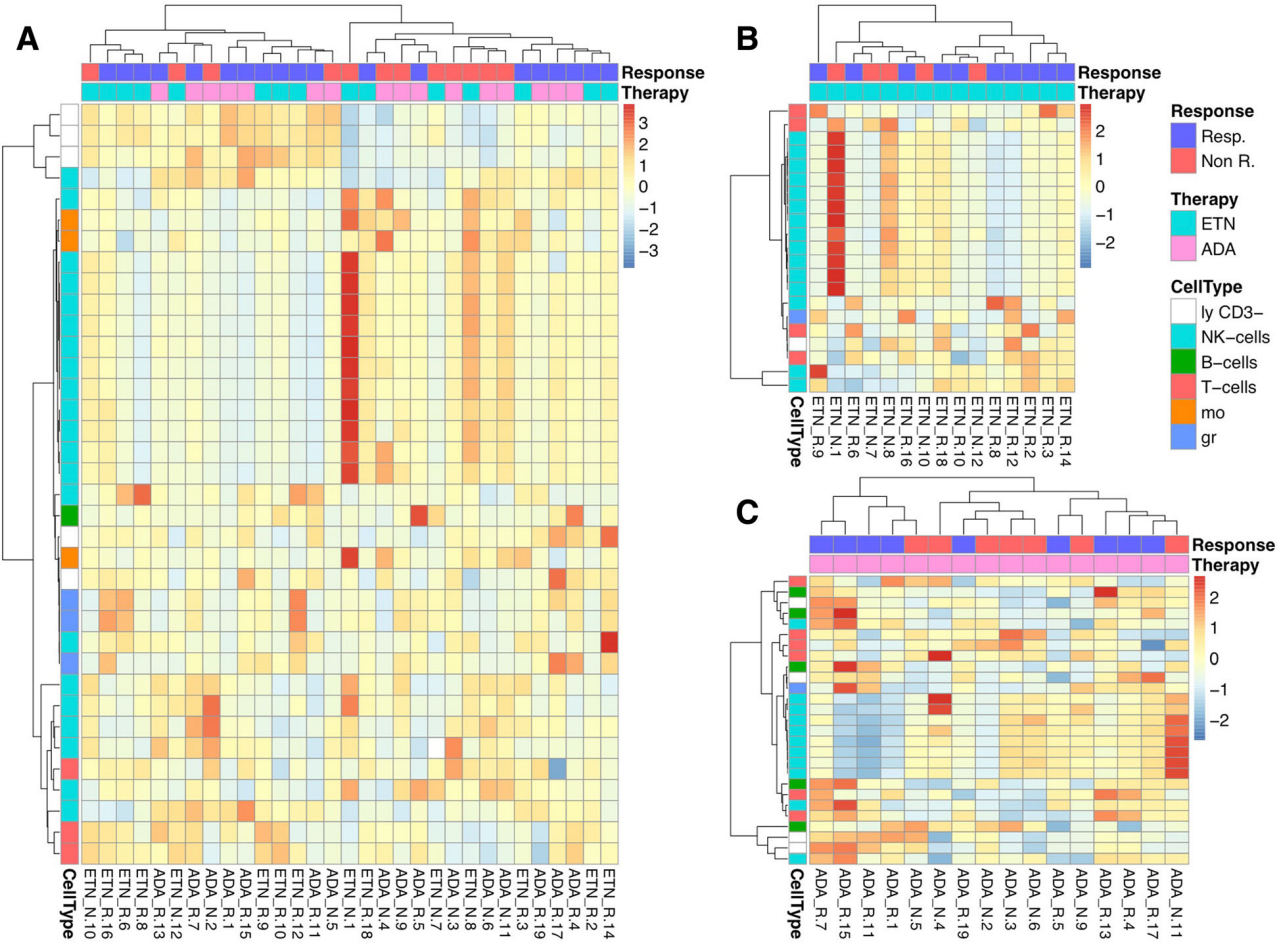
Two-dimensional hierarchical cluster analyses of immunophenotypic parameters that are differentially expressed in R and NR prior to treatment when considering all patients (**a**), etanercept (ETN) alone (**b**), and adalimumab (ADA) alone (**c**). Columns represent individual patients and the colour code below the vertical dendrogram indicates responders (Resp.; blue) or non-responders (Non R.; red) and the TNF-blocker used (either ETN (cyan) or ADA (pink)). Rows of the cluster diagram represent immunophenotypic parameters with *p* values < 0.1. The magnitude of parameter expression is colour coded with red for a relatively increased and blue for a relatively decreased expression. The colour code for the horizontal dendrogram indicates the expression in a particular cell type, such as natural killer (NK) cells (cyan), B cells (green), T cells (raspberry-red), monocytes (mo; orange), granulocytes (gr; blue), and CD3-negative lymphocytes (ly CD3–; white). In total, one million cells were acquired per sample to ensure that even rare cell populations with frequencies around 0.1% could be reliable detected

The presented two-dimensional clustering approach with patients clustered in the vertical direction and immunophenotypic parameters clustered in the horizontal direction gives an overview of all leukocyte subsets including certain activation markers that are differentially expressed in R and NR. For the identification of significant parameters by the immunoClust algorithm, FCS files (Flow Cytometry Standard file format) of uncompensated raw data were used and finally disclosed 36 parameters when all patient samples were considered. Analysing ETN- and ADA-treated patients separately revealed 21 and 27 parameters, respectively (Fig. 1).

Although using all these parameters did not allow an error-free classification of R and NR, all samples were grouped into two main clusters which were enriched for R and NR, respectively (Fig. 1a). Surprisingly, more than 50% of the discriminating parameters could be clearly assigned to NK cell subsets if all patients were analysed together (Fig. 1a). For further analysis of NK cell-related subsets, and knowing that ETN and ADA have different modes of action to neutralise the effect of TNF-α, we continued to investigate both treatment groups separately to identify therapy-specific response signatures. Using this approach, the majority of parameters that significantly discriminate between NR and R in the ETN group (Fig. 1b) and ADA group (Fig. 1c) were related to the NK cell compartment. The best classification of R and NR was achieved in the group of ETN-treated patients. Here, only two of 10 R were grouped as NR and one single of five NR was grouped as R.

### Validation of classical NK cells and CD8-positive NK cells as potential immunological biomarkers for an anti-TNF-α therapy prediction

**Fig. 2:**
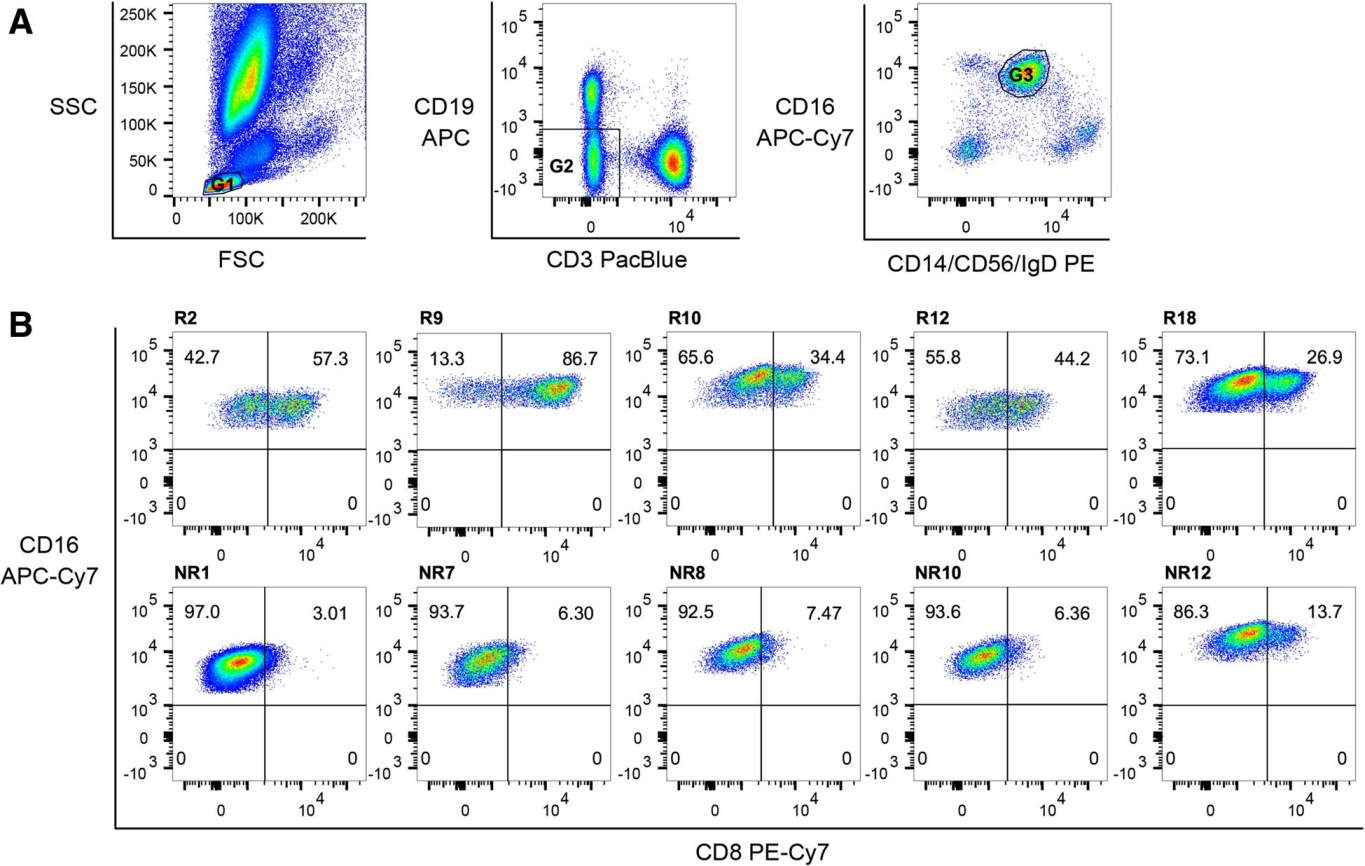
Representative gating strategy to identify CD8-positive and CD8-negative NK cell subsets. To increase parameter diversity detectable in a single seven-colour staining setup we have combined specifically expressed cell lineage markers that are labelled by the same fluorochrome, such as CD14-PE/CD56-PE/IgD-PE. Its cell-specific expression can be deconvoluted by sequential gating as exemplified by **a**: (left panel) gating on small cells (G1) with low granularity according to FSC/SSC characteristics allows exclusion of monocytes (CD14) and granulocytes (CD16); (middle panel) exclusion of B lymphocytes by CD19 and T lymphocytes by CD3 (G2); and (right panel) CD19/CD3-double negative cells were analysed for CD56 versus CD16 allowing us to identify NK cells by the co-expression of CD56 and CD16 (G3). **b** Classical NK cells were further analysed according to their expression of the CD8 receptor in five exemplary responders (R; top row) and non-responders (NR; bottom row)

Since both the percentage distribution of classical NK cells in general and the ratio of CD8-positive and CD8-negative NK cells in particular appeared to be the most promising predictors for an anti-TNF therapy response, we looked at the CD8 receptor expression on NK cells in more detail. In Fig. 2 the general gating strategy to define classical CD56^dim^CD16^+^ NK cells is shown. In Additional file 2 (Figure S1), backgating for monocytes and NK cells demonstrates that, despite labelling multiple antigens by the same fluorochrome (CD14/CD56/IgD labelled to PE), unravelling of these complex stainings with respect to NK cells, monocytes, T cells, and B cells is possible.

At first, a lymphocyte scatter gate (G1) was set (Fig. 2a). Next T and B cells were excluded out of the lymphocyte population (G2) to determine the percentage of CD56^dim^CD16^+^ NK cells (G3). Subsequently, the CD8-positive NK cells were quantified by sub-gating as shown exemplarily for five responders (R2, R9, R16, R12, and R18) and five non-responders (NR1, NR7, NR8, NR10, and NR12) (Fig. 2b).

**Fig. 3:**
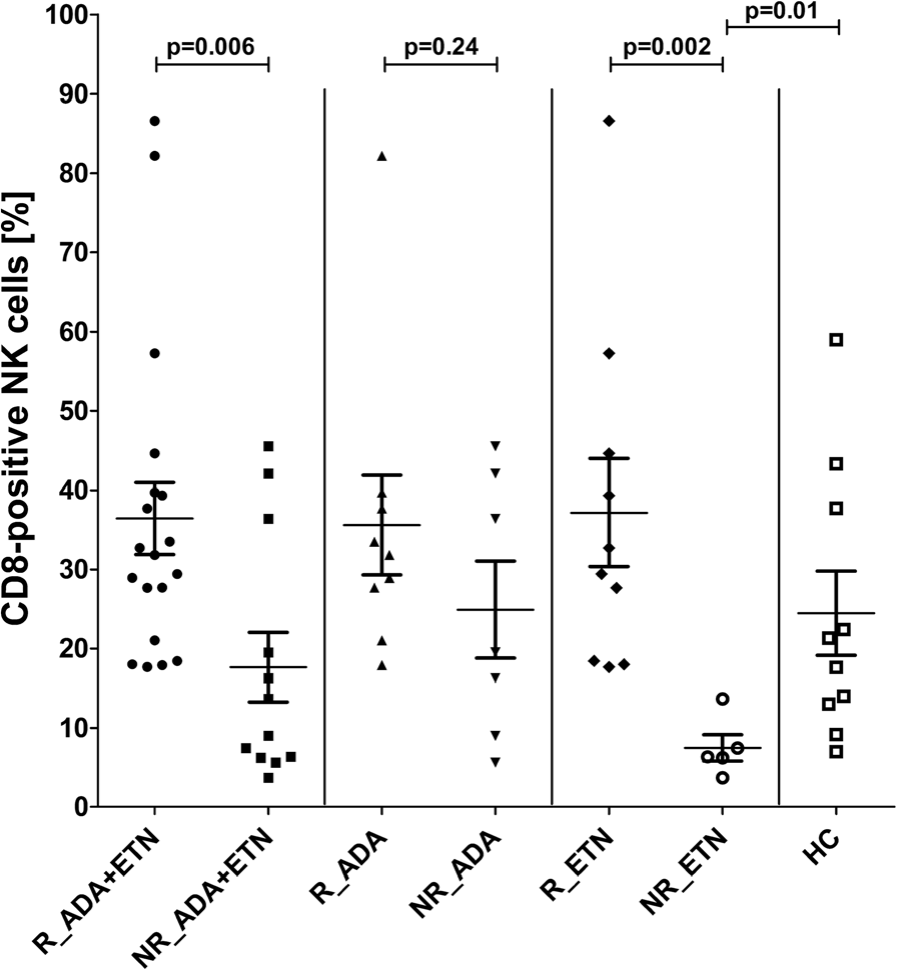
Frequencies of CD8-positive natural killer (NK) cells in responders (R) and non-responders (NR) assessed before treatment for all patients or for adalimumab (ADA) and etanercept (ETN) separately. Significance was determined by Welch’s corrected *t* test. HC healthy controls

Figure 3 shows the frequencies of CD8-positive NK cells for healthy controls (*n* = 10), anti-TNF R (*n* = 19), and anti-TNF NR (*n* = 12). A significantly higher frequency of CD8 receptor-bearing NK cells was observed in the R group compared with NR (Fig. 3). Comparing the percentage of CD8-positive NK cells to HC, AS patients who will not respond showed significantly lower frequencies of CD8-expressing NK cells (Fig. 3). Individual values for frequencies and absolute counts of NK cells and CD8-positive subsets are given in Additional file 1 (Table S1).

**Fig. 4:**
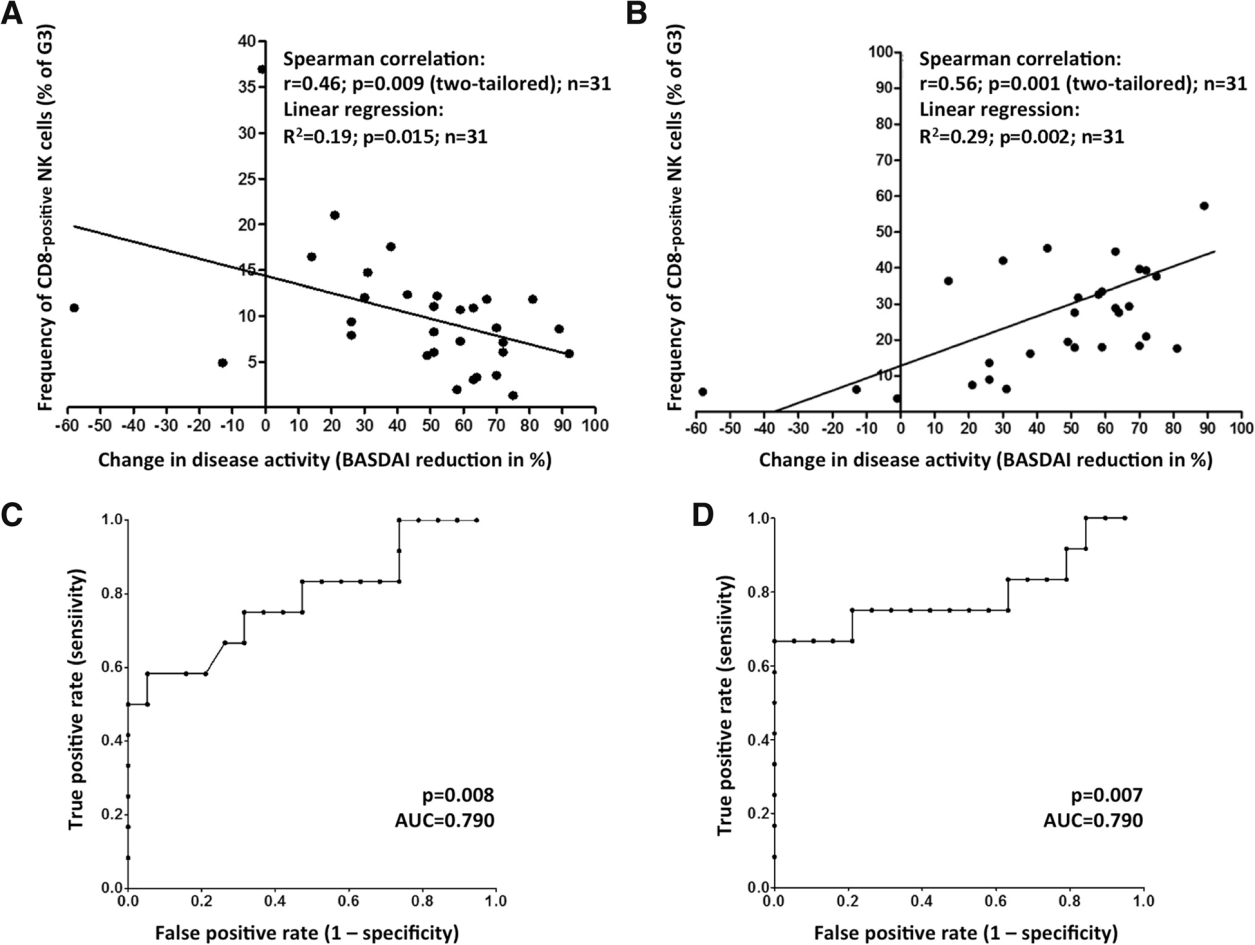
Linear regression and Spearman’s rank correlation analyses showing relative reduction of Bath Ankylosing Spondylitis Disease Activity Index (BASDAI) values within 1 to 6 months after treatment with ADA and ETN (*n* = 31), associated with changes in frequencies of classical natural killer (NK) cells (**a**) and CD8-positive NK cells (**b**). ROC curves for all patients demonstrating the value of classical (**c**) and CD8-expressing NK cells (**d**) as prognostic markers according to increasing area under the curve (AUC) values

Next, we performed Spearman’s rank correlation and linear regression analyses which showed a significant inverse correlation of frequencies of classical NK cells and an improved therapy outcome if all 31 patients were included (Fig. 4a; *p* = 0.01, *r*^2^ = 0.19).

Surprisingly, a positive, linear correlation was found when we focused on the analysis of CD8- expressing NK cells (Fig. 4b). Here, the frequency of CD8-positive cells related to the cells of the total NK cells (G3) clearly correlated with a successful therapy response. This correlation was statistically significant when all patients were considered (*p* = 0.002, *r*^2^ = 0.29).

Furthermore, reverse regression analyses, displayed by ROC curves, were performed to verify the quality of classical and especially CD8-positive NK cells as suitable cellular biomarkers for predicting an anti-TNF therapy outcome (Fig. 4c, d). If all samples were included for this analysis, the frequency of classical NK cells was the most promising parameter as a baseline predictor (Fig. 4c; *p* = 0.008, area under the curve (AUC) = 0.79). Slightly improved values were obtained if the frequency of CD8-positive NK cells was used (Fig. 4d; *p* = 0.007, AUC = 0.79). Therefore, our data implicate that the appearance of CD8-positive NK cells is a robust biomarker for the prediction of an anti-TNF-α response at baseline.

## Discussion

To our knowledge, this is the first study aiming to identify cellular biomarkers in peripheral blood to stratify AS patients upfront with regard to subsequent responsiveness to anti-TNF-α therapy. To date, there are no quantifiable laboratory parameters available that can be used for a personalised response prediction [34]. Although higher values of CRP, ESR, and serum amyloid A (SAA), together with the presence of HLA-B27, have been reported to be useful baseline predictors for a successful anti-TNF-α therapy response in AS, the robustness, sensitivity, and specificity fail if applied to individual patients [35, 36]. Even our data, such as the age of patients, disease duration, CRP levels, or blood sedimentation, did not allow any prediction of future responsiveness.

Other potential new biomarkers described, such as single nucleotide polymorphisms (SNPs) and activity of endoplasmic reticulum aminopeptidase (ERAP)-1 [37], serum levels of matrix metalloproteinase (MMP)-3 [38], or vascular endothelial growth factor (VEGF) [39], have not yet reached a standard in the clinical diagnostic routine of AS. Generally, it is challenging to correlate quantifiable parameters to the disease activity BASDAI score, which is calculated on the basis of a patient’s subjective assessment of well-being and therefore is only of limited value when used as an absolute variable.

In our explorative study presented here we used an integrated multi-parametric flow cytometry and a new unsupervised data clustering approach to identify possible cellular biomarkers that are qualitatively or quantitatively different in responders and non-responders. A similar approach has been successfully used to classify active AS patients from healthy donors according to specific phenotypical changes in blood [32]. A comprehensive overview about immunophenotypical changes described so far in different autoimmune diseases is given by Alegria et al. [40].

Principally, at first view, an increased frequency of the major CD56^dim^CD16^+^ NK cell subset was indicative for a weak therapeutic anti-TNF response. This finding is in line with other reports showing that innate immunity and particularly NK cells may play a central role in the pathogenesis of various autoimmune diseases both in a protective and pathogenic manner. Their frequency and functionality were investigated in chronic inflammation, such as rheumatoid arthritis [41], multiple sclerosis [42], psoriasis [43], and systemic lupus erythematosus [44]. Despite some conflicting results, an overall decrease and a cytolytic impairment of circulating NK cells could be observed. In AS, the functionality of NK cells is described to be likewise impaired [45] but, in contrast to other autoimmune diseases, an increased NK cell number is reported [46, 47]. We could confirm this observation if comparing the non-responder group with normal donors (Fig. 3a); however, the NK cell frequency in the responder cohort was similar to the number of healthy individuals. The strong association between NK cells and the aetiology of AS is underlined by the capability of the disease-dependent elevated killer cell immunoglobulin-like receptors (KIRs) to recognise the HLA-B27 antigen, which is expressed in 80–90% of AS patients [48, 49]. Concomitant with a pathogenetic upregulated frequency of KIR3DL1^+^ NK cells in AS, interferon (IFN)γ production is correspondingly diminished [50]. Moreover, activated KIR3DL2^+^ NK cells are increased in SpA and may play a pathogenic role.

An in-depth analysis of the NK cell compartment revealed that the frequency of classical NK cells expressing the CD8 antigen showed a significant and positive correlation with anti-TNF responsiveness. It is known that about 40% of NK cells variably express CD8 in an α/α homodimeric form whereas the CD8-positive subset is described to exhibit enhanced cytotoxic features as compared with its CD8-negative counterpart [51, 52]. We could not detect significant age- or sex-related differences with respect to the frequency of CD8-positive NK cells either in the group of healthy controls or in that of AS patients. Thus, our data imply that NK cells expressing the CD8αα homodimer are directly or indirectly involved in the immunosuppressive effect exerted by anti-TNF-α blockers.

If CD8αα-expressing NK cells are directly involved it can be assumed that their increased cytotoxic activity and/or their diminished behaviour regarding cytotoxicity-induced apoptosis are responsible for the improved responsiveness of TNF blockers. Alternatively, it can be hypothesised that an engagement of CD8αα receptors on NK cells to molecules of the HLA-I family can cause an increased secretion of the pro-inflammatory cytokines TNF-α and IFNγ [53], which in turn promote both TNF receptor (TNFR) synthesis and its proteolysis to a soluble form [54]. By this mechanism, endogenously synthesised sTNFRs [55, 56] can co-operatively support the action of therapeutic TNF inhibitors [57, 58]. These non-signalling ‘decoy’ receptors are still competent for binding TNF and thus may function as a natural TNF antagonist comparable to ETN [59]. Since we have included adalimumab- and etanercept-treated patients in our study, it was interesting to know if differences in the prediction of responsiveness were detectable when either TNF-α was neutralised by ADA or scavenged by the sTNFR2 fusion protein (ETN). Although group size reduction was responsible for a less statistical power of prediction analysis, we could ascertain a better correlation with respect to ETN-treated patients compared with ADA-treated patients. Therefore, these findings are encouraging for validation in new independent cohorts of appropriate group sizes. If this result could be validated it would indicate diverse and more complex modes of action going beyond the mere neutralising effect of TNF-α blockers.

To elucidate possible differences in the TNFR expression in CD8-positive and CD8-negative NK cell subsets isolated from healthy individuals, we performed global gene expression analyses but could not detect any differential expression of TNFR1, TNFR2, or TNF-α (data not shown). Comparing the expression of TNFR1 and TNFR2 in healthy individuals, it was obvious that higher expression levels were detectable for TNFR2 (data not shown). Unfortunately, thus far we have had no opportunity for analysing cells isolated from AS patients to test if TNFRs or TNF-α were differentially expressed under chronic inflammatory conditions. Thus, according to our data, it can be postulated that CD8-positive NK cells are obviously capable of amplifying the neutralising effect of TNF-blockers, but it remains unclear why this amplification is preferentially observed in ETN- and not in ADA-treated patients.

## Conclusions

Although our findings are promising, a further validation of CD8-positive NK cells as a potential biomarker for TNF responsiveness is necessary in an independent cohort of AS and other rheumatic and gastrointestinal diseases where anti-TNF blockers are successfully administered. Nevertheless, our study is a first proof of the concept that cellular response signatures can be identified in peripheral blood by an extensive immunophenotyping approach. Even though the exact mechanism of how CD8-positive NK cells and the therapeutic effect of TNF-α blockers are interrelated in AS is only poorly understood thus far, monitoring these cells by flow cytometry offers an interesting new diagnostic option with respect to the challenges of an individualised therapy concept, at least in the field of chronic inflammatory rheumatic diseases.

## Additional files

Additional file 1: **Table S1.** Individual data on treatment, responsiveness, NK cell counts per μl blood, and frequencies related to total leukocytes. Moreover, absolute numbers and frequencies of CD8-positive NK cells in relation to all CD56/CD16-double positive NK cells are given. n.d. not determined. (DOCX 25 kb)

Additional file 2: **Figure S1.** Clarification of the gating strategy for NK cells shown in Fig. 2. By backgating, monocytes can be clearly separated from NK cells (Gate A, PBMC; Gate B, CD3/CD19-double negative PBMC; Gate C, monocytes; and Gate D, NK cells). (DOC 87 kb)

## Abbreviations

ADA: Adalimumab
AS: Ankylosing spondylitis
BASDAI: Bath Ankylosing Spondylitis Disease Activity Index
CRP: C-reactive protein
ERAP: Endoplasmic reticulum aminopeptidase
ESR: Erythrocyte sedimentation rate
ETN: Etanercept
HC: Healthy controls
IBD: Inflammatory bowel disease
JIA: Juvenile inflammatory arthritis
KIR: Killer cell immunoglobulin-like receptor
MFI: Mean fluorescence intensity
MMP: Matrix metalloproteinase
NK: Natural killer
NR: Non-responders
NSAID: Non-steroidal anti-inflammatory drug
Pso: Psoriasis
R: Responders
RA: Rheumatoid arthritis
SAA: Serum amyloid A
SNP: Single nucleotide polymorphism
SpA: Spondyloarthritis
sTNFR: Soluble human tumour necrosis factor receptor
TNF: Tumour necrosis factor
VEGF: Vascular endothelial growth factor
