# Supplementary material for: An explorative study on deep profiling of peripheral leukocytes to identify predictors for responsiveness to anti-tumour necrosis factor alpha therapies in ankylosing spondylitis: natural killer cells in focus
Source: Arthritis Res Ther. 2018 Aug 29;20:191. doi: 10.1186/s13075-018-1692-y (PMC6116509; doi:10.1186/s13075-018-1692-y)
Supplement: Supplementary file 1 — Table S1. Individual data on treatment, responsiveness, NK cell counts per μl blood, and frequencies related to total leukocytes. Moreover, absolute numbers and frequencies of CD8-positive NK cells in relation to all CD56/CD16-double positive NK cells are given. n.d. not determined. (DOCX 25 kb) [file 13075_2018_1692_MOESM1_ESM.docx]

Additional file 1: Table S1 This table summarizes individual data on treatment, responsiveness, NK cell counts per µl blood and frequencies related to total leukocytes. Moreover, absolute numbers and frequencies of CD8-positive NK cells in relation to all CD56/CD16-double positive NK cells are given. n.d. not determined
