# Supplementary material for: An explorative study on deep profiling of peripheral leukocytes to identify predictors for responsiveness to anti-tumour necrosis factor alpha therapies in ankylosing spondylitis: natural killer cells in focus
Source: Arthritis Res Ther. 2018 Aug 29;20:191. doi: 10.1186/s13075-018-1692-y (PMC6116509; doi:10.1186/s13075-018-1692-y)

Additional file 2: Figure S1 To clarify the gating strategy for NK cells shown in figure 2 we included this supplementary figure S1, which shows by backgating that monocytes can be clearly separated from NK cells (Gate A: PBMC; Gate B: CD3/CD19-double negative PBMC ; Gate C: monocytes and Gate D: NK cells).


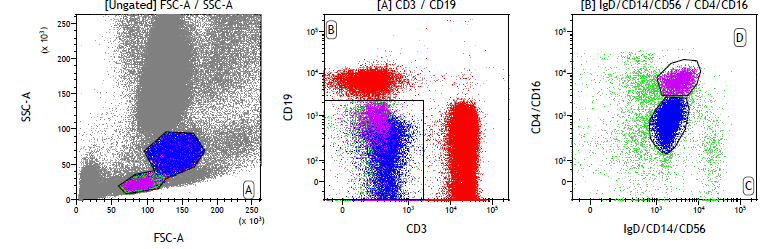

Supplement: Supplementary file 2 — Figure S1. Clarification of the gating strategy for NK cells shown in Fig. 2. By backgating, monocytes can be clearly separated from NK cells (Gate A, PBMC; Gate B, CD3/CD19-double negative PBMC; Gate C, monocytes; and Gate D, NK cells). (DOC 87 kb) [file 13075_2018_1692_MOESM2_ESM.doc]
